# Sequentially inducible mouse models reveal that *Npm1* mutation causes malignant transformation of *Dnmt3a*-mutant clonal hematopoiesis

**DOI:** 10.1038/s41375-018-0368-6

**Published:** 2019-01-28

**Authors:** Matthew A. Loberg, Rebecca K. Bell, Leslie O. Goodwin, Elizabeth Eudy, Linde A. Miles, Jennifer M. SanMiguel, Kira Young, David E. Bergstrom, Ross L. Levine, Rebekka K. Schneider, Jennifer J. Trowbridge

**Affiliations:** 1 The Jackson Laboratory, Bar Harbor, ME USA; 2 Memorial Sloan Kettering Cancer Center, New York, NY USA; 3 Department of Hematology, Erasmus MC Cancer Institute, Rotterdam, The Netherlands

**Keywords:** Cancer models, Haematopoietic stem cells, Cancer stem cells, Acute myeloid leukaemia, Myeloproliferative disease

## Abstract

Clonal hematopoiesis (CH) is a common aging-associated condition with increased risk of hematologic malignancy. Knowledge of the mechanisms driving evolution from CH to overt malignancy has been hampered by a lack of in vivo models that orthogonally activate mutant alleles. Here, we develop independently regulatable mutations in DNA methyltransferase 3A (*Dnmt3a*) and nucleophosmin 1 (*Npm1*), observed in human CH and AML, respectively. We find *Dnmt3a* mutation expands hematopoietic stem and multipotent progenitor cells (HSC/MPPs), modeling CH. Induction of mutant *Npm1* after development of *Dnmt3a*-mutant CH causes progression to myeloproliferative disorder (MPD), and more aggressive MPD is observed with longer latency between mutations. MPDs uniformly progress to acute myeloid leukemia (AML) following transplant, accompanied by a decrease in HSC/MPPs and an increase in myeloid-restricted progenitors, the latter of which propagate AML in tertiary recipient mice. At a molecular level, progression of CH to MPD is accompanied by selection for mutations activating Ras/Raf/MAPK signaling. Progression to AML is characterized by additional oncogenic signaling mutations (*Ptpn11*, *Pik3r1*, *Flt3*) and/or mutations in epigenetic regulators (*Hdac1*, *Idh1*, *Arid1a*). Together, our study demonstrates that *Npm1* mutation drives evolution of *Dnmt3a*-mutant CH to AML and rate of disease progression is accelerated with longer latency of CH.

## Introduction

Clonal hematopoiesis (CH) occurs when stem and progenitor cell clones gain one or more somatic mutations that confer a competitive advantage [1]. This can be observed in patients by an increase in the variant allele frequency (VAF) of somatic mutations in the blood or bone marrow (BM) [2]. There is a vast spectrum of outcomes in individuals with CH; in many cases, CH causes little to no clinically relevant hematopoietic phenotype. At the other extreme, CH cases represent a permissive state for development of BM failure syndromes including myelodysplastic syndrome (MDS) [3, 4], myeloproliferation including myeloproliferative disorder (MPD) [5], or leukemias including acute myeloid leukemia (AML), the last of which is accompanied by additional somatic mutations [6–8]. Pressing clinical issues include how to identify individuals with CH for which this condition should be considered a medically important event and how to develop therapies that might prevent onset of hematologic malignancies. To answer these questions, we need to understand the factors (specific somatic mutations, genetic background of the individual, environmental factors, etc.) that cause evolution of CH to hematologic malignancies. A current barrier is the lack of available animal models that undergo the evolution of CH to hematologic malignancies in a native in vivo environment.

The most common somatic mutations present in CH are in genes associated with epigenetic modifications, including the DNA methyltransferase *DNMT3A*, and splicing factors. Several mouse models of *DNMT3A*-driven CH have been generated by conditional knockout [9], mutant allele knockin [10, 11], or mutant allele overexpression [12]. Further, several studies have identified cooperating mutations that can act synergistically with *Dnmt3a* knockout or mutation to cause leukemia, such as *Flt3*^ITD^ [13, 14], *Flt3*^ITD^ and *Npm1*^c^ [11], *cKit* [15], and *Kras*^G12D^ [16]. However, existing combinatorial mutation models are limited by the use of germline alleles with conditional alleles (thereby not modeling the known somatic nature of these mutations), multiple conditional alleles that are induced simultaneously by Cre recombination, or conditional alleles combined with ex vivo transduction/overexpression of cooperating mutations. Thus, based on the design of previous mouse models, none are able to evaluate the evolution of CH to hematologic malignancies within a native in vivo environment.

To overcome these limitations, we have developed an inducible, dual-recombinase system combining flippase-FRT (Flp-FRT) and Cre-loxP recombination technologies [17, 18] to improve genetically engineered mouse models of CH and to allow study of the evolution of CH to hematopoietic malignancy. This dual-recombinase system allows for sequential induction of a *Dnmt3a* hotspot mutation in R878H (*Dnmt3a*^R878H^), replicating human R882H [1], and the recurrent hotspot mutation in the multifunctional nuclear protein *Npm1* (*Npm1*^c^ type A; *Npm1*^cA^) [19, 20]. Previously, concurrent expression of *Dnmt3a*^R878H^ and *Npm1*^cA^ mutations was not found to cause a lethal malignancy within a 45-week observation period [11]; however, it remains unknown whether malignancy would have occurred over a longer timeframe or with a latency period between expression of these two mutations. Therefore, we have utilized our model to examine the cellular and molecular alterations during evolution of CH to hematologic malignancy within a native in vivo environment.

## Materials and methods

### Genetic engineering of *Dnmt3a*^fl-R878H/+^ and *Npm1*^frt-cA/+^ mice

Targeting vectors mDnmt3a_LSL_exon23_bGHpA_pBlight and mNpm1_FS12_exon11_FSF_pBlight were generated by in vitro synthesis (GenScript). The *Dnmt3a*^fl-R878H/+^ construct design placed wild-type exon 23 in a loxP-flanked STOP cassette (4xSV40pA) into the intron between exons 22 and 23, and the 3′ homology arm included the 2633 G>A in exon 23 to encode the R878H mutation. The *Npm1*^frt-cA/+^ construct design placed wild-type exon 11 in an FRT-flanked STOP cassette (4xSV40pA) into the intron between exons 10 and 11, and the 3’ homology arm included a frameshift mutation to create a humanized mutant exon 11 (p.W288fs*12). Constructs were linearized and electroporated into C57BL/6 embryonic stem (ES) cells. Clones were screened by loss-of-allele PCR, confirmed by Southern blot and microinjected into C57BL/6 blastocysts. PCR genotyping of pups was used to screen for germline transmission (primer sequences in Table S1).

### Experimental animals

C57BL/6J and B6.SJL-*Ptprc*^*a*^*Pepc*^*b*^/BoyJ (referred to as “B6.CD45.1”) mice were obtained from, and aged within, The Jackson Laboratory. *Dnmt3a*^fl-R878H/+^ mice were crossed to B6.Cg-Tg(Mx1-cre)1Cgn/J mice (referred to as *Mx1*-Cre) [21]. *Npm1*^frt-cA/+^ mice were crossed to B6N.129S6(Cg)-*Gt(ROSA)26Sor*^*tm3(CAG-flpo/ERT2)Alj*^/J (referred to as *R26*^FlpoER^) [22]. Unless otherwise noted, all mice were female and experiments initiated at 8–16 weeks of age. Mice were injected five times (once every other day) via intraperitoneal (IP) injection with 15 mg/kg high molecular weight polyinosinic-polycytidylic acid (pIpC) (InvivoGen) to induce *Mx1*-Cre recombinase expression. Mice were administered 125 mg/kg tamoxifen three times (on consecutive days) via oral gavage to induce *R26*^FlpoER^ recombinase expression. Before and after pIpC or tamoxifen administration, genomic DNA was extracted from PB cells for recombination PCR using primers specified above (Table S1). In addition, RNA was extracted and cDNA synthesized from whole BM cells for Sanger sequencing to verify mutant allele expression. Real-time PCR was utilized to examine relative expression of *Dnmt3a* in *Dnmt3a*^fl-R878H/+^ *Mx1*-cre^+^ animals prior to pIpC injection (primer sequences in Table S1). To verify cellular localization of Npm1^cA^, spleens from *Npm1*^cA/+^ *R26*^FlpoER^ or control *Npm1*^+/+^ *R26*^FlpoER^ mice were fixed, paraffin embedded, and sectioned for immunohistochemistry using a polyclonal antibody raised against human NPM1 (Cell Signaling Technology). The Jackson Laboratory’s Institutional Animal Care and Use Committee (IACUC) approved all experiments.

### Primary cell isolation and hematopoietic stem/progenitor cell phenotyping

Single-cell suspensions of BM were prepared by filtering crushed, pooled femurs, tibiae, and iliac crests from each mouse. BM mononuclear cells (MNCs) were isolated by Ficoll-Paque (GE Healthcare Life Sciences) density centrifugation and stained with a combination of fluorochrome-conjugated antibodies from eBioscience, BD Biosciences, or BioLegend: CD45.2 (clone 104), c-Kit (clone 2B8), Sca-1 (clone 108129), CD150 (clone TC15-12F12.2), CD48 (clone HM48-1), FLT3 (Clone A2F10), CD34 (clone RAM34), FcγR (clone 2.4G2), mature lineage (Lin) marker mix (B220 (clone RA3-6B2), CD11b (clone M1/70), CD4 (clone RM4-5), CD8a (clone 53-6.7), Ter-119 (clone TER-119), Gr-1 (clone RB6-8C5)), and the viability stain propidium iodide (PI). Stained cells were either analyzed or sorted using a FACSAria with Diva software (BD Biosciences) based on the following surface marker profiles: LSK (Lin^−^ Sca-1^+^ c-Kit^+^), LT-HSC (Lin^−^ Sca-1^+^ c-Kit^+^ Flt3^−^ CD150^+^ CD48^−^), ST-HSC (Lin^−^ Sca-1^+^ c-Kit^+^ Flt3^−^ CD150^−^ CD48^−^), MPP2 (Lin^−^ Sca-1^+^ c-Kit^+^ Flt3^−^ CD150^+^ CD48^+^), MPP3 (Lin^−^ Sca-1^+^ c-Kit^+^ Flt3^−^ CD150^−^ CD48^+^), MPP4 (Lin^−^ Sca-1^+^ c-Kit^+^ Flt3^+^), myeloid-restricted progenitors (MyPro; Lin^−^ Sca-1^−^ c-Kit^+^), GMP (Lin^−^ Sca-1^-^ c-Kit^+^ CD150^−^ CD34^+^ FcγR^+^), CMP (Lin^−^ Sca-1^−^ c-Kit^+^ CD150^−^ CD34^+^ FcγR^lo^) and MEP (Lin^−^ Sca-1^−^ c-Kit^+^ CD150^−^ CD34^−^ FcγR^−^). All flow cytometry data was analyzed using FlowJo software.

### Colony formation unit (CFU) assay

De novo isolated cells were plated in MethoCult GF M3434 (StemCell Technologies) at the indicated numbers and cultured at 37 °C and 5% CO_2_. Colonies were scored between 6 and 14 days post-plating using a Nikon Eclipse TS100 inverted microscope. For CFU replating assay, colonies were harvested, total viable cell counts obtained, and then 5 × 10^4^ cells were replated in MethoCult GF M3434. To examine allele recombination at the clonal level, whole BM was plated from *Dnmt3a*^R878H/+^ *Npm1*^cA/+^ mice with MDS/MPD or MPD phenotypes. Individual colonies were picked under a Nikon Eclipse microscope (4× magnification) and used for colony PCR to examine recombination of *Dnmt3a*^R878H^ and *Npm1*^cA^ alleles (primers listed in Table S1).

### In vivo transplantation

For competitive transplantation, 2 × 10^6^ BM MNCs from *Dnmt3a*^fl-R878H/+^ *Mx1*-Cre or *Dnmt3a*^+/+^ *Mx1*-Cre mice were combined at a 1:1 ratio with 2 × 10^6^ BM MNCs from B6.CD45.1 mice and intravenously injected into female recipient B6.CD45.1 mice that received a lethal dose of gamma irradiation (1200 rads, split dose). Recipient mice received pIpC injections as described at 5 weeks post-transplant. For non-competitive transplantation, 10^6^ BM MNCs from *Dnmt3a*^R878H/+^ *Npm1*^cA/+^ *Mx1*-Cre *R26*^FlpoER^ or *Dnmt3a*^+/+^ *Npm1*^+/+^ *Mx1*-Cre *R26*^FlpoER^ mice were intravenously injected into female recipient B6.CD45.1 mice that received a lethal dose of gamma irradiation (1200 rads, split dose). Recipient mice received pIpC injections as described at 4–5 weeks post-transplant, and tamoxifen administration at various times post-pIpC as indicated. Recipient mice were monitored every 4 weeks thereafter by flow cytometry analysis of PB samples using a cocktail of CD45.1 (clone A20), CD45.2, CD11b, B220, CD3ε (clone 145-2C11), and Gr-1 on an LSRII (BD). For secondary transplantation studies, female B6.CD45.1 mice received a sublethal dose of gamma irradiation (600 rads) and were intravenously injected with 10^6^ BM MNCs from moribund primary recipients of *Dnmt3a*^R878H/+^ *Npm1*^cA/+^ *Mx1*-Cre *R26*^FlpoER^ cells. For tertiary transplantation studies, female B6.CD45.1 mice received a sublethal dose of gamma irradiation (600 rads) and were intravenously injected with 5 × 10^3^ sorted MyPro (Lin^−^ Sca-1^−^ c-Kit^+^) from moribund secondary recipients of *Dnmt3a*^R878H/+^ *Npm1*^cA/+^ *Mx1*-Cre *R26*^FlpoER^ cells.

### Analysis of moribund mice

Mice demonstrating declining health status were sacrificed and PB, spleen, liver, and BM harvested. Differential blood cell counts were obtained from PB using an Advia 120 Hematology Analyzer (Siemens). Single-cell suspensions of PB, spleen, and BM were analyzed by flow cytometry for mature lineage markers and c-Kit on an LSRII. Cytospin preparations of whole BM MNCs were stained with May–Grunwald–Giemsa stain. Liver and spleens were fixed for 24 h in 10% buffered formalin phosphate, embedded in paraffin, and sections were stained with H&E. Images of stained BM, liver, and spleen slides were captured on a Nikon Eclipse E200 upright microscope with SPOT imaging software (v.5.2).

### M-IMPACT

Genomic DNA was extracted from isolated BM cells of diseased mice and WT littermates. DNA underwent targeted capture and deep sequencing performed by the MSKCC Integrated Genomics Operation Core using the MSK mouse Integrated Mutation Profiling of Actionable Cancer Targets (M-IMPACT) v1 assay. M-IMPACT is a DNA sequencing assay, which captures and sequences the exons and select introns of 578 known cancer genes, using solution-phase hybridization-based exon capture as described previously [23]. Gene selection, mouse ortholog mapping, and bait design has been described previously [23, 24]. Mouse genomic DNA (250 ng) was used for library construction with molecular barcoding of each sample occurring prior to capture and sequencing. Equimolarly pooled libraries containing captured DNA fragments were subsequently sequenced on one lane of an Illumina HiSeq system for paired end 125/125 reads with intended 500× coverage for all reads. Sequencing analysis was performed by the MSKCC Bioinformatics Core. Processed FASTQ files were mapped to the mouse reference genome mm10 (GRCm38) using BWA-MEM (Burrows-Wheeler Aligner v0.7.12; http://arxiv.org/abs/1303.3997). Resulting files were sorted, grouped, and PCR duplicates identified by MarkDuplicates in PICARD Tools (v1.124; https://github.com/broadinstitute/picard). BAM files were processed through GATK toolkit (v3.2) [25] to perform variant calling in tumor-vs-normal paired mode. Specifically, somatic single-nucleotide variants (SNVs) were called from processed BAM files using muTect (v1.1.7) [26]. Haplotype caller from GATK was used to identify somatic small insertions and deletions (indels) with a custom post-processing script (https://github.com/soccin/Variant-PostProcess). Variant allele frequency (VAF) was determined by calculating the fraction of variant reads for a specific out of the total reads at that location.

### Statistical analysis

No sample group randomization or blinding was performed. For overall survival, Log-rank (Mantel–Cox) test was performed on Kaplan–Meier survival curves. Statistical analysis of non-survival data was performed by unpaired student’s *t*-test, non-parametric one-way ANOVA (Kruskal–Wallis test) followed by Dunn’s multiple comparisons test, or two-way ANOVA followed by Tukey’s multiple comparisons test. For these experiments, a minimum of three samples per condition was chosen to detect statistical differences with >80% power at an error rate of 5%. All statistical tests, including evaluation of normal distribution of data and examination of variance between groups being statistically compared, were assessed using Prism 7 software (GraphPad).

## Results

### Generation of an independently regulatable *Dnmt3a*^R878H^ allele

**Fig. 1:**
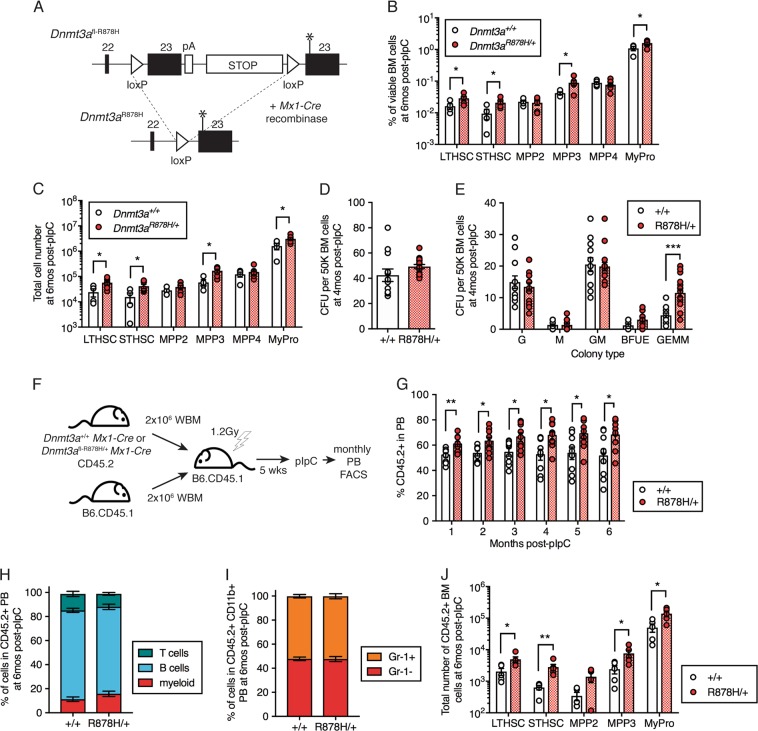
*Dnmt3a*^R878H/+^ expands HSCs and multipotent progenitor cells. **a** Schematic diagram of the design of the *Dnmt3a*^fl-R878H/+^ allele. Asterisk indicates 2633G>A in exon 23 to encode the R878H mutation. **b** Frequency and **c** total number of LT-HSC, ST-HSC, MPP2, MPP3, MPP4, and MyPro cells in the BM of +/+ (*n* = 4) and R878H/+ (*n* = 6) mice at 6 months post-pIpC. **d** Total colony-forming units (CFU) and **e** colony types derived from 50 K BM MNCs isolated from +/+ (*n* = 6) and R878H/+ (*n* = 8) mice at 4 months post-pIpC. G (granulocyte), M (macrophage), GM (granulocyte-macrophage), BFUE (burst forming-unit erythroid), GEMM (mixed granulocyte-erythroid-macrophage-megakaryocyte). Results are from three independent experiments. **f** Experimental design for competitive transplantation of +/+ or fl-R878H/+ BM cells with wild-type B6.CD45.1 BM cells at a 1:1 ratio into lethally irradiated B6.CD45.1 recipient mice followed by pIpC to induce recombination of *Dnmt3a*^fl-R878H^. **g** Frequency of donor-derived (CD45.2^+^)+/+ (*n* = 9) or R878H/+ (*n* *=* 9) cells in PB of recipient mice post-pIpC. Results are from two independent experiments. **h** Frequency of myeloid (CD11b^+^), B (B220^+^), and T (CD3^+^) cells within donor-derived (CD45.2^+^) PB and **i** Gr-1^+^ and Gr-1^−^ cells within donor-derived myeloid PB at 6 months post-pIpC. **j** Frequency of LT-HSC, ST-HSC, MPP2, MPP3, and MyPro cells within donor-derived +/+ (*n* = 5) or R878H/+ (*n* = 5) BM at 6 months post-pIpC. Results are from two independent experiments. In all graphs, dots represent individual mice, bars indicate mean ± s.e.m. *P < 0.05; **P < 0.01; ***P < 0.001

To generate an inducible mouse model of human *DNMT3A-*mutant (*DNMT3A*^R882H^) CH, we engineered a Cre-inducible knockin model of the equivalent *Dnmt3a*^R878H^ mutation in mice (Fig. 1a, Fig. S1A,B). While the general targeting strategy was similar to two recently published knockin models of *Dnmt3a*^R878H^ [10, 11], our model has unique advantages of preserving expression of wild-type *Dnmt3a* prior to *Mx1*-Cre-mediated recombination (Fig. S1C) and following recombination, the *Dnmt3a*^R878H^ mutant allele retains endogenous polyA and 3′ UTR elements. Indeed, prior to induction of *Mx1*-Cre by injection of poly(I:C) (pIpC), there is no observable change in frequency of the hematopoietic stem and progenitor cell compartment in the BM of *Dnmt3a*^fl-R878H^ mice (Fig. S1D). Furthermore, there is no observable change in frequency of stem and progenitor cell subsets including long-term HSCs (LT-HSC), short-term HSCs (ST-HSC), multipotent progenitor cell 2 (MPP2), MPP3, and MPP4 in the BM of *Dnmt3a*^fl-R878H^ mice (Fig. S1E, gating strategy shown in Fig. S1F). Thus, our mice model somatic acquisition of *Dnmt3a* mutation as it is observed in human CH.

### *Dnmt3a*^R878H/+^ expands the HSC and MPP compartments

As *Dnmt3a* mutation is considered to be an early event causing CH, we first investigated the phenotype of our *Dnmt3a*^R878H/+^ *Mx1-Cre* mice. At 6 months following activation of Cre recombinase expression, we observed a significant expansion of both the percentage and total number of viable LT-HSC, ST-HSC, MPP3, and myeloid progenitors (MyPro) in *Dnmt3a*^R878H/+^ vs. control *Dnmt3a*^+/+^ mice (Fig. 1b, c). To examine the clonogenic potential of *Dnmt3a*^R878H/+^ cells, we isolated whole BM cells from control and *Dnmt3a*^R878H/+^ mice for colony-forming unit (CFU) assays. We observed similar numbers of total CFU emerging from control and *Dnmt3a*^R878H/+^ mice (Fig. 1d). However, there was a significant expansion of multi-lineage, mixed granulocyte/erythroid/macrophage/megakaryocyte (GEMM) colonies (Fig. 1e), consistent with an expansion of multipotent stem and progenitor cells in the BM. Together, this data is generally consistent with human data demonstrating that the HSC compartment is most dramatically altered by the *Dnmt3a*^R882H^ somatic mutation [8] and existing mouse *Dnmt3a*^R878H^ models demonstrating expansion of stem and progenitor cells [10, 11].

To test the competitive fitness of *Dnmt3a*^R878H/+^ LT-HSCs, we examined their ability to competitively reconstitute the hematopoietic system of an irradiated host. We isolated BM cells from control and *Dnmt3a*^R878H/+^ mice that had not yet received pIpC and transplanted them at a 1:1 ratio with wild-type B6.CD45.1 BM cells into lethally irradiated congenic B6.CD45.1 recipient mice (Fig. 1f). At 5 weeks post-transplant, following initial homing and engraftment of transplanted LT-HSCs, all recipients were administered pIpC to induce *Mx1*-Cre expression and followed by monthly peripheral blood (PB) flow cytometry analysis. We observed that the chimerism (% CD45.2^+^ cells) in the PB was significantly higher from *Dnmt3a*^R878H/+^ cells compared to control *Dnmt3a*^+/+^ cells (Fig. 1g), supporting a competitive advantage of *Dnmt3a*^R878H/+^ HSCs. To determine whether this chimerism was biased toward production of particular types of mature hematopoietic cells, we examined myeloid (CD11b^+^), B cell (B220^+^) and T cell (CD3^+^) populations, and Gr-1^+^ and Gr-1^−^ cells (within the myeloid fraction) of donor-derived CD45.2^+^ PB cells. We observed no differences in mature hematopoietic lineage composition between *Dnmt3a*^R878H/+^ and control *Dnmt3a*^+/+^ cells (Fig. 1h, i). In contrast, examination of chimerism within the hematopoietic stem and progenitor cell compartment of recipient mice revealed increased numbers of donor-derived LT-HSC, ST-HSC, MPP3, and myeloid-restricted progenitors (MyPro) (Fig. 1j). As this result closely resembles the hematopoietic stem and progenitor cell expansion in non-transplanted mice (Fig. 1c), these data together indicate that this expansion is a cell-intrinsic effect of the *Dnmt3a*^R878H/+^mutation.

### Generation of an independently regulatable *Npm1*^cA^ allele

**Fig. 2:**
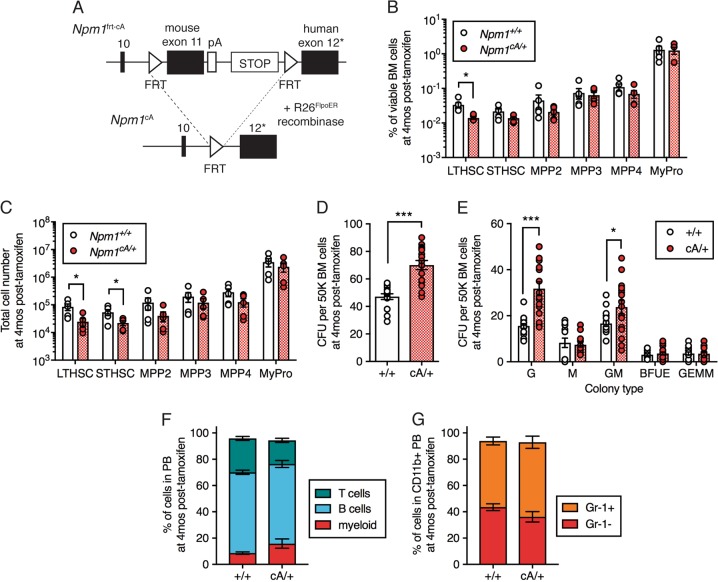
*Npm1*^cA/+^ depletes HSCs and expands myeloid colony-forming cells. **a** Schematic diagram of the design of the *Npm1*^frt-cA/+^ allele. Asterisk indicates frameshift mutation to create a humanized mutant exon 12 (p.W288fs*12). **b** Frequency and **c** total number of LT-HSC, ST-HSC, MPP2, MPP3, MPP4, and MyPro cells in the BM of +/+ (*n* = 5) and cA/+ (*n* = 5) mice at 4 months post-tamoxifen. **d** Total CFU and **e** colony types derived from 50K BM MNCs isolated from +/+ (*n* = 8) and cA/+ (*n* = 8) mice at 4 months post-tamoxifen. Results are from four independent experiments. **f** Frequency of myeloid, B, and T cells within PB and **g** Gr-1^+^ and Gr-1^−^ cells within myeloid PB at 4 months post-tamoxifen in +/+ (*n* = 6) and cA/+ (*n* = 12) mice. In all graphs, dots represent individual mice, bars indicate mean ± s.e.m. *P < 0.05; ***P < 0.001

To develop an independently regulatable cooperating mutation that may synergize with *Dnmt3a*^R878H^ to drive AML, we generated a Flp-inducible knockin mouse model of humanized *Npm1*^cA^ (Fig. 2a, Fig. S2A–C). Mutations involving the *NPM1* are found in one-third of all AML cases [20]. *NPM1* mutations (referred to as *NPM1*^c^) disrupt tryptophan residues and generate an additional nuclear export signal leading to aberrant localization of NPM1^c^ in the cytoplasm [27]. Our general targeting strategy was similar to a published knockin model of humanized *Npm1*^flox-cA^ [28]; however, our model has a unique advantage of being Flp recombinase-inducible by the use of FRT sites rather than loxP sites. This strategy allows for independent regulation of the *Dnmt3a* and *Npm1* mutant alleles. We utilized a *Rosa26*-knockin tamoxifen-inducible *FlpoER* mouse strain [22] to induce expression of *Npm1*^cA^ following administration of tamoxifen. To compare alterations in the hematopoietic stem and progenitor cell compartment driven by *Npm1*^cA^ as a single mutation with the *Dnmt3a*^R878H/+^ phenotype, we examined the frequency and total number of stem and progenitor cells in *Npm1*^cA/+^ mice at 4 months post-tamoxifen injection. This revealed a significant decrease in frequency of LT-HSC (Fig. 2b) as well as total numbers of LT-HSC and ST-HSC (Fig. 2c) in *Npm1*^cA/+^ compared to *Npm1*^+/+^ control mice. To examine the clonogenic potential of *Npm1*^cA/+^ cells, we isolated whole BM cells from control and *Npm1*^cA/+^ mice at 4 months post-tamoxifen injection for CFU assays. We observed a significant increase in the total number of colonies formed (Fig. 2d) and expansion in both granulocyte (G) and granulocyte-macrophage (GM) colony types (Fig. 2e). This *Npm1*^cA/+^ in vitro phenotype is in contrast to the *Dnmt3a*^R878H/+^ BM, which demonstrated an expansion of the multi-lineage GEMM colony type (Fig. 1e). Lastly, as *Npm1*^cA/+^ has been demonstrated to cause myeloproliferation and AML with 30% penetrance late in life (median survival 617 days) [28], we examined mature hematopoietic cell lineage composition in the PB in our *Npm1*^cA/+^ mice at 4 months post-tamoxifen injection. We observed no alteration in frequency of mature hematopoietic lineages in *Npm1*^cA/+^ mice compared to controls (Fig. 2f, g), suggesting that at the time point at which we observe changes in the hematopoietic stem and progenitor cell compartment and clonogenicity of BM cells, there is no overt peripheral myeloproliferation.

### *Dnmt3a*^R878H/+^*Npm1*^cA/+^ mice develop a lethal myeloproliferative disorder

**Fig. 3:**
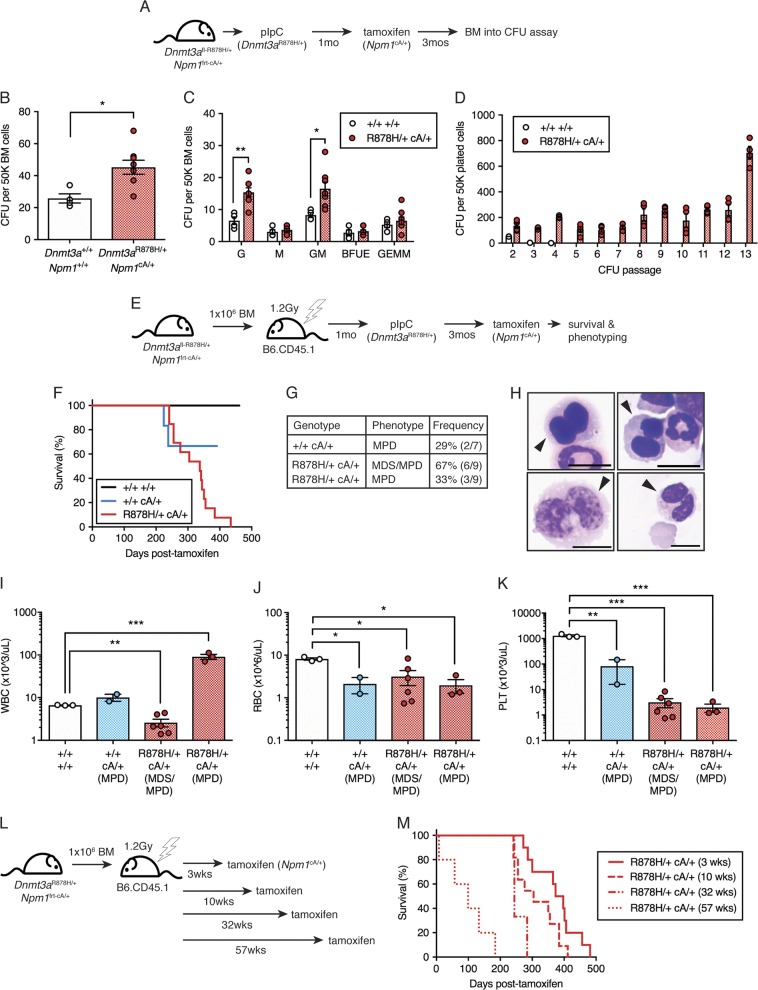
*Dnmt3a*^R878H/+^ followed by *Npm1*^cA/+^ causes development of lethal MPD. **a** Experimental design for induction of *Dnmt3a*^R878H/+^ driving clonal hematopoietic expansion prior to induction of *Npm1*^cA/+^, followed by *in vitro* CFU assay. **b** Total CFU and **c** colony types derived from 50K BM MNCs isolated from control (*n* = 4) or R878H/+ cA/+ (*n* *=* 8) mice at 3 months post-tamoxifen. Results are from 3 independent experiments. (**d**) CFU derived from 50K BM MNCs isolated from control (*n* = 2) and R878H/+ cA/+ (*n* *=* 4) mice, serially passaged for up to 13 passages. Results are from two independent experiments. **e** Experimental design for non-competitive transplantation of fl-R878H/+ frt-cA/+ BM into lethally irradiated recipient mice, followed by pIpC induction of R878H/+ and clonal hematopoietic expansion prior to tamoxifen induction of cA/+. **f** Overall survival of mice transplanted with 10^6^ BM cells from control (*n* = 3), cA/+ only (*n* = 7) or R878H/+ cA/+ (*n* = 13) mice. Results are from three independent experiments. **g** Description and penetrance of phenotypes in moribund mice following transplant with cA/+ only or R878H/+ cA/+ BM cells. **h** Representative Giemsa-stained BM cytospins (100×) from R878H/+ cA/+ MDS/MPD showing dysplastic cells (arrows). Scale bars are 10 μm. **i** WBC count, **j** RBC count, and **k** PLT count in moribund mice (control, *n* *=* 3; cA/+ MPD, *n* = 2; R878H/+ cA/+ MDS/MPD, *n* = 6; R878H/+ cA/+ MPD, *n* = 3). **l** Experimental design for tamoxifen induction of cA/+ at various times following induction of R878H/+. **m** Overall survival of mice transplanted with 10^6^ BM cells with latency between R878H/+ and cA/+ mutations of 3 weeks (*n* = 10), 10 weeks (*n* = 11), 32 weeks (*n* *=* 3), or 57 weeks (*n* = 5). Results are from three independent experiments. In all graphs, dots represent individual mice, bars indicate mean ± s.e.m. *P < 0.05; **P < 0.01; ***P < 0.001

We then crossed the *Npm1*^frt-cA/+^ mice with the *Dnmt3a*^fl-R878H/+^ *Mx1-Cre* model, to generate compound *Dnmt3a*^fl-R878H/+^ *Npm1*^frt-cA/+^ *Mx1-Cre R26*^FlpoER^ mice. We induced expression of the *Dnmt3a*^R878H^ mutation first then followed this with induction of *Npm1*^cA^ expression three months later to allow development of CH in the interim period (Fig. 3a). We first examined the clonogenic potential of BM cells isolated from these *Dnmt3a*^R878H/+^ *Npm1*^cA/+^ mice. We observed a significant increase in total CFU from *Dnmt3a*^R878H/+^ *Npm1*^cA/+^ BM compared to control (Fig. 3b). Assessing colony morphology revealed a significant increase in granulocyte (G) and granulocyte-macrophage (GM) colony types (Fig. 3c). Serial replating of *Dnmt3a*^R878H/+^ *Npm1*^cA/+^ colonies revealed robust proliferative and clonogenic potential for at least 13 passages (Fig. 3d), suggesting unlimited in vitro self-renewal potential.

To determine whether this enhanced clonogenic potential was reflective of transformation, we transplanted BM from control *Dnmt3a*^+/+^ *Npm1*^+/+^, single mutant *Npm1*^frt-cA/+^ or double mutant *Dnmt3a*^fl-R878H/+^ *Npm1*^frt-cA/+^ mice into lethally irradiated recipient mice, administered pIpC at 1-month post-transplant, tamoxifen at 3 months post-pIpC, and followed the natural life course of the recipients (Fig. 3e). Importantly, this experimental design restricted expression of *Dnmt3a*^R878H^ and *Npm1*^cA^ exclusively to the hematopoietic compartment. We observed that within a 440-day period, 100% of *Dnmt3a*^R878H/+^ *Npm1*^cA/+^ recipient mice died while only 29% of single mutant *Npm1*^cA/+^ recipient mice died (Fig. 3f). The phenotypes at the time of death comprised two major groups; 67% (6 out of 9) *Dnmt3a*^R878H/+^ *Npm1*^cA/+^ mice had mixed myelodysplastic syndrome and myeloproliferative disorder (MDS/MPD) while 33% (3 out of 9) *Dnmt3a*^R878H/+^ *Npm1*^cA/+^ mice and 29% (2 out of 7) single mutant *Npm1*^cA/+^ mice had MPD (Fig. 3g). The single mutant *Npm1*^cA/+^ mice developed MPD with survival times of ~7.5–8 months post-tamoxifen, demonstrating that this mutation is capable of causing myeloproliferation beyond our original 4-month post-tamoxifen sampling (Fig. 2f).

An extensive characterization of *Dnmt3a*^R878H/+^ *Npm1*^cA/+^ MDS/MPD, *Dnmt3a*^R878H/+^ *Npm1*^cA/+^ MPD and single mutant *Npm1*^cA/+^ MPD was performed to contrast phenotypic similarities and differences. The *Dnmt3a*^R878H/+^ *Npm1*^cA/+^ MDS/MPD phenotype was characterized by erythroid and myeloid dysplasia (Fig. 3h), pancytopenia including significantly decreased white blood cell (WBC) (Fig. 3i), red blood cell (RBC) (Fig. 3j), and platelet (PLT) counts (Fig. 3k), increased PB myeloid cell production (Fig. S3A), and myeloid cell infiltration into the spleen and liver (Fig. S3C,E). The *Dnmt3a*^R878H/+^ *Npm1*^cA/+^ MPD phenotype was characterized by significantly increased WBC count (Fig. 3i), significantly decreased RBC and PLT counts (Fig. 3j, k), significantly increased spleen weight (Fig. S3D), increased PB myeloid cell production with monocyte bias (Fig. S3A,B), and myeloid (monocyte) cell infiltration into the spleen and liver (Fig. S3C–F). The single mutant *Npm1*^cA/+^ MPD phenotype was distinguished from *Dnmt3a*^R878H/+^ *Npm1*^cA/+^ MPD phenotype by lower WBC count (Fig. 3i), lower spleen weight (Fig. S3D) and greater monocyte bias in myeloid PB cells (Fig. S3B). Together, these data demonstrate that acquiring the *Npm1*^cA^ mutation on pre-existing *Dnmt3a*^R878H/+^ CH causes progression to MDS/MPD or MPD.

To demonstrate one application of independently regulatable disease alleles, we examined whether the duration of pre-existing *Dnmt3a*^R878H/+^ CH would impact disease development or survival of mice. *Npm1*^cA/+^ was induced at 3, 10, 32, or 57 weeks post-recombination of *Dnmt3a*^R878H/+^ (Fig. 3l). We observed that longer latency between acquisition of *Dnmt3a* and *Npm1* mutations resulted in a significantly shorter survival time post-tamoxifen (3 vs. 10 weeks, *P* = 0.067; 3 vs. 32 weeks, ***P = 0.0009; 3 vs. 57 weeks, ***P < 0.0001) (Fig. 3m). This suggests that a greater length of time with *Dnmt3a*-mutant clonal expansion increases the risk of progression to malignancy.

### *Dnmt3a*^R878H/+^*Npm1*^cA/+^ MPD progresses to AML upon transplant

**Fig. 4:**
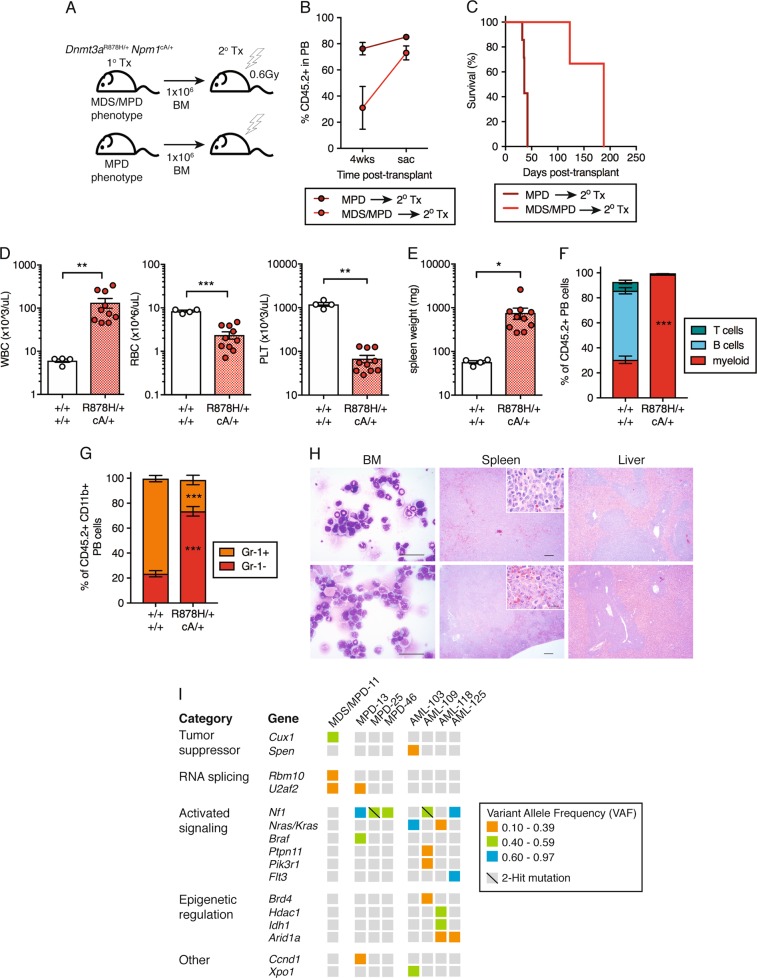
*Dnmt3a*^R878H/+^ *Npm1*^cA/+^ MPD progresses to AML following transplantation. **a** Experimental design for non-competitive secondary transplantation of *Dnmt3a*^R878H/+^ *Npm1*^cA/+^ BM MNCs from primary recipient mice with MDS/MPD or MPD phenotypes. **b** Frequency of donor-derived (CD45.2^+^) cells in PB of secondary recipient mice at 4 weeks post-transplant and at time of sacrifice. Dots represent mean ± s.e.m. (MPD, *n* = 7; MDS/MPD, *n* = 3). Results are from three independent experiments. **c** Overall survival of secondary transplant-recipient mice (MPD, *n* = 7; MDS/MPD, *n* = 3). **d** WBC, RBC, and PLT counts and **e** spleen weights of moribund mice (control, *n* = 4; +/+ cA/+, *n* = 10). **f** Frequency of myeloid, B, and T cells within the donor-derived CD45.2^+^ fraction in PB and **g** Gr-1^+^ and Gr-1^−^ cells within donor-derived myeloid PB of moribund mice (control, *n* = 3; +/+ cA/+, *n* *=* 10). **h** Representative Giemsa-stained BM cytospins (far left; 40×, scale bars are 40 μm) and H&E-stained spleen (center; 4×, scale bars are 100 μm) (inset: 40×, scale bars are 40 μm) and liver sections (far right; 4×, scale bars are 100 μm) from moribund recipient mice. **i** Somatic, nonsynonymous mutations in individual genes and sets of genes, grouped into five categories. Orange boxes indicate mutations at VAF 0.10-0.39; green boxes, VAF 0.40–0.59; blue boxes, VAF 0.60–0.97. In all graphs unless otherwise specified, dots represent individual mice, bars indicate mean ± s.e.m. *P < 0.05; **P < 0.01; ***P < 0.001

While human *NPM1* mutations are most often observed in *de novo* AML, several studies have shown that *NPM1* mutations can be present in MDS or MDS/myeloproliferative neoplasm (MPN) prior to blast counts reaching the threshold for AML diagnosis [29, 30]. This suggests *Dnmt3a*^R878H/+^ *Npm1*^cA/+^ MDS/MPD or MPD might have potential for further progression to AML. To determine whether *Dnmt3a*^R878H/+^ *Npm1*^cA/+^ MPDs were transplantable to recipient mice and to test whether they are capable of further progression to AML, we isolated BM cells from primary *Dnmt3a*^R878H/+^ *Npm1*^cA/+^ transplant recipients with either the MDS/MPD or MPD phenotypes and transplanted these cells into sublethally irradiated secondary recipient mice (Fig. 4a). We assessed donor cell engraftment (% CD45.2^+^) at 4 weeks post-transplant as well as when these mice were moribund. This analysis revealed that MPD samples achieved high levels of engraftment in secondary recipients almost immediately post-transplant (Fig. 4b). In contrast, MDS/MPD samples had initially low levels of engraftment in secondary recipients but dramatically expanded to achieve high levels of engraftment at the time of sacrifice. There was a significant difference between the survival of mice transplanted with MDS/MPD donors (median survival 188 days) vs. MPD donors (median survival 36 days) (Fig. 4c). However, these both represent a dramatic disease acceleration compared to the primary recipient animals (Fig. 3f).

We observed that 100% of secondary transplant recipient mice succumbed to acute myeloid leukemia (AML), regardless of whether they were derived from an MDS/MPD or MPD primary donor. All *Dnmt3a*^R878H/+^ *Npm1*^cA/+^ recipients showed significantly increased WBC count and decreased RBC and PLT counts (Fig. 4d), increased spleen weight (Fig. 4e), complete myeloid cell dominance in the PB with monocyte bias (Fig. 4f, g), >20% blast cells in the BM and vast infiltration of blast cells in the spleen and liver (Fig. 4h). Together these data suggest that *Dnmt3a*^R878H/+^ *Npm1*^cA/+^ MDS/MPD and MPD can both progress to AML.

To examine clonality of recombination of *Dnmt3a*^fl-R878H^ and *Npm1*^frt-cA^ alleles in mice with MDS/MPD, MPD and AML, we performed a BM CFU assay and picked individual colonies for PCR. We observed that in MDS/MPD, 82% of colonies had recombination of both *Dnmt3a*^R878H^ and *Npm1*^cA^ (Fig. S3G). In MPD and AML, 100% of colonies had recombination of both *Dnmt3a*^R878H^ and *Npm1*^cA^. This demonstrates similar levels of recombination in *Dnmt3a*^R878H/+^ *Npm1*^cA/+^ MDS/MPD, MPD, and AML. To further examine additional acquired somatic mutations, we performed somatic mutational analysis of BM from MDS/MPD, MPD and AML samples using a custom targeted sequencing panel querying 578 known cancer genes (MSK-Mouse IMPACT; M-IMPACT) (Table S2). We observed that *Dnmt3a*^R878H/+^ *Npm1*^cA/+^ MDS/MPD was associated with mutations in the tumor suppressor *Cux1*, previously shown to drive MDS and MDS/MPD [31] and in the RNA splicing genes *Rbm10* and *U2af2* (Fig. 4i, Table S3). Mutations in key factors of the spliceosome are known to occur frequently in CH and MDS [32]. Development of *Dnmt3a*^R878H/+^ *Npm1*^cA/+^ MPD was associated with mutations activating the Ras/Raf/MAPK pathway (including *Nf1*, *Nras*, *Kras*, and *Braf*) at high VAF, consistent with known function of these mutations [33, 34]. Progression to *Dnmt3a*^R878H/+^ *Npm1*^cA/+^ AML was also associated with these same mutations, but with the addition of mutations activating the tyrosine phosphatase SHP2 (*Ptpn11*), PI3K (*Pik3r1*), or Flt3 (*Flt3*) signaling as well as mutations involved in epigenetic regulation including *Brd4*, *Hdac1*, *Idh1*, and *Arid1a*. Many of these genes are recurrently mutated in human AML [35–37], and demonstrate additional mutations are required to cooperate with mutant *Dnmt3a* and *Npm1* to promote transformation to MDS/MPD, MPD and AML.

### *Dnmt3a*^R878H/+^*Npm1*^cA/+^ AML mice have decreased HSC and increased myeloid progenitors

**Fig. 5:**
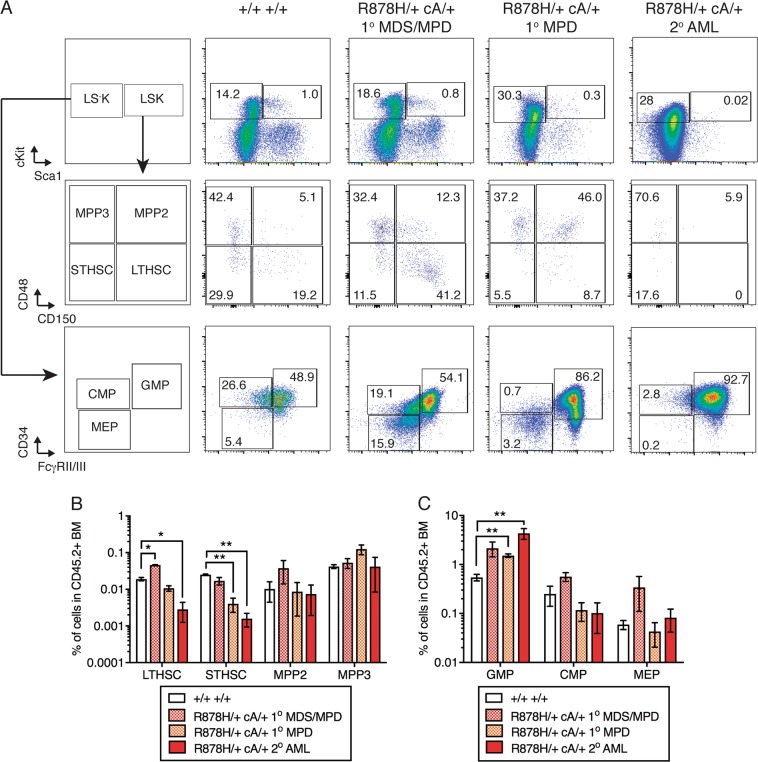
Mice with *Dnmt3a*^R878H/+^ *Npm1*^cA/+^ AML show expansion of granulocyte-macrophage progenitor cells. **a** Representative flow cytometric gating for hematopoietic stem and progenitor cells in recipient mice transplanted with *Dnmt3a*^+/+^ *Npm1*^+/+^ or *Dnmt3a*^R878H/+^ *Npm1*^cA/+^ with MDS/MPD, MPD, or AML phenotypes. **b** Frequency of LT-HSC, ST-HSC, MPP2, and MPP3 and **c** GMP, CMP, and MEP in donor-derived CD45.2^+^ BM cells (control, *n* = 2; R878H/+ cA/+ MDS/MPD, *n* = 2; R878H/+ cA/+ MPD, *n* = 3; R878H/+cA/+ AML, *n* = 4). Results are from two independent experiments

To determine how *Dnmt3a*^R878H/+^ CH progresses to MPD and AML with acquisition of *Npm1*^cA^ mutation, we examined the BM stem and progenitor cell compartment of primary and secondary recipient mice transplanted with *Dnmt3a*^R878H/+^ *Npm1*^cA/+^ cells (representative gating shown in Fig. 5a). We observed a significant increase in LTHSC frequency in the *Dnmt3a*^R878H/+^ *Npm1*^cA/+^ MDS/MPD group and decrease in LT-HSC frequency in the *Dnmt3a*^R878H/+^ *Npm1*^cA/+^ AML group compared to control transplanted mice (Fig. 5b). In addition, we observed a significant decrease in ST-HSC frequency in the *Dnmt3a*^R878H/+^ *Npm1*^cA/+^ MPD and AML groups compared to control transplanted mice. Strikingly, we observed that granulocyte-macrophage progenitors (GMP) were significantly expanded in the *Dnmt3a*^R878H/+^ *Npm1*^cA/+^ MPD and AML groups vs. the control group (Fig. 5c). Together, these data suggest that *Npm1*^cA^ transformation of *Dnmt3a*^R878H/+^ CH is accompanied by a shift in the stem and progenitor cell compartment towards expansion of myeloid-restricted progenitor cells at the expense of HSCs.

### *Dnmt3a*^R878H/+^*Npm1*^cA/+^ AML is propagated by myeloid-restricted progenitors

**Fig. 6:**
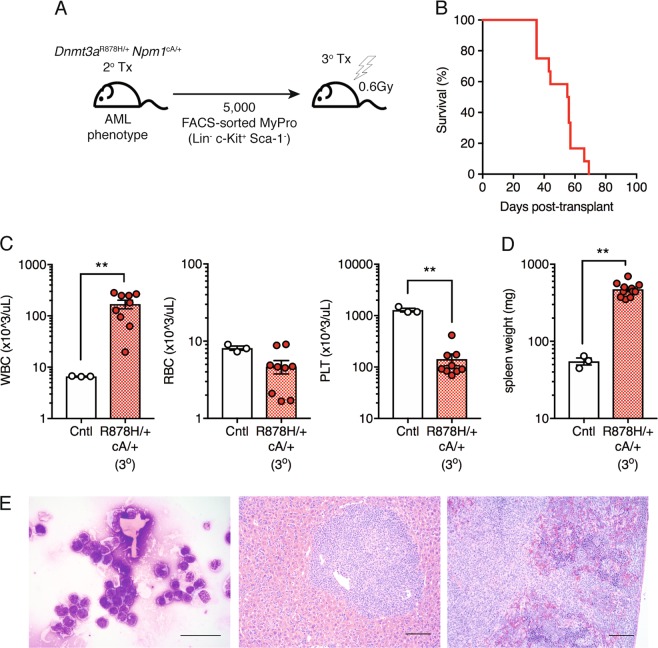
Myeloid-restricted progenitor cells are tumor-propagating cells in *Dnmt3a*^R878H/+^ *Npm1*^cA/+^ AML. **a** Experimental design for non-competitive tertiary transplantation of FACS-sorted *Dnmt3a*^R878H/+^ *Npm1*^cA/+^ BM myeloid progenitors from secondary recipient mice with AML. **b** Overall survival of tertiary transplant recipient mice (*n* = 12). Results are from three independent experiments. **c** WBC, RBC, and PLT counts in moribund mice (control, *n* = 3; +/+ cA/+, *n* = 9). **d** Spleen weights of moribund mice (control, *n* = 3; +/+ cA/+, *n* = 11). **e** Representative Giemsa-stained BM cytospin (far left; 40×, scale bars are 40 μm) and H&E-stained spleen (center; 10×, scale bars are 100 μm) and liver sections (far right; 10×, scale bars are 100 μm) from moribund recipient mice. In all graphs, dots represent individual mice, bars indicate mean ± s.e.m. **P < 0.01

Within *Dnmt3a*^R878H/+^ *Npm1*^cA/+^ AML, the shift in the stem and progenitor cell compartment towards expansion of myeloid-restricted progenitor cells suggests that these cells may represent tumor-propagating cells capable of transferring disease to recipient mice. To test this, we performed a tertiary transplant of sorted myeloid progenitors (Lin^−^ c-Kit^+^ Sca-1^−^) (Fig. 6a). We observed that 100% of tertiary transplant recipient mice succumbed to acute myeloid leukemia (AML) with a median survival of 56 days (Fig. 6b), similar to the rate and penetrance of AML development in secondary transplant recipients (Fig. 4c). This is evidenced in all recipients by significantly increased WBC and decreased PLT (Fig. 6c) counts, increased spleen weight (Fig. 6d), >20% blast cells in the BM and vast infiltration of blast cells in the spleen and liver (Fig. 6e). Together, these data support diagnosis of AML in secondary recipient animals and demonstrate that myeloid-restricted progenitors are leukemia stem cells (LSCs) capable of propagating *Dnmt3a*^R878H/+^ *Npm1*^cA/+^ AML.

## Discussion

Individuals with CH have an increased risk of development of hematologic malignancy, including AML. Currently, no methods exist to distinguish individuals with benign CH from those with pre-malignant CH due to a lack of understanding of the mechanisms by which CH progresses to AML. Improved in vivo models of the progression of CH to AML are needed to identify specific pathways and molecules for development of prognostic tests and therapies to prevent progression to malignancy. In this study, we have developed novel inducible mouse models allowing temporal control of expression of *Dnmt3a*^R878H^, a common event in human CH, and *Npm1*^cA^, a common cooperating mutation in human AML. We find that acquisition of *Npm1*^cA/+^ mutation on the background of pre-existing *Dnmt3a*^R878H/+^ CH drives development of MPD, which is transformed to AML upon subsequent transplantation. Furthermore, increasing the duration of *Dnmt3a*^R878H/+^ CH increased the aggressiveness of MPD development upon acquisition of *Npm1*^cA/+^ mutation and decreased overall survival in mice. Previously, the combination of *Dnmt3a*^R878H/+^ and *Npm1*^cA/+^ induced simultaneously did not cause lethal hematologic malignancy within a 45-week timeframe [11]. We hypothesize that this is distinct from our current findings based on the timeframe of the observation period of animals and the sequential acquisition of mutations over time with concomitant genetic and epigenetic evolution. Based on our results, we propose that accumulation of cellular and molecular alterations over time, as a consequence of *Dnmt3a* mutation, synergize with *Npm1* mutation to cause more aggressive malignancy. These data are pertinent for considering the timing and importance of follow-up mutational screening in individuals with CH.

In our CH model, we find that heterozygous *Dnmt3a*^R878H^ alone is sufficient to cause expansion of phenotypic and functional HSCs and MPPs, and confer a competitive advantage of LTHSCs over wild-type LTHSCs in vivo. Integrating this phenotype with published models of *Dnmt3a* knockout, which demonstrate specific expansion of LTHSCs [9], or *Dnmt3a*^R878H^ mutation, which demonstrate expansion in both multipotent and myeloid progenitors downstream of the LTHSC [10, 11], suggest that the *Dnmt3a*^R878H^ mutation is distinct from *Dnmt3a* loss. *Dnmt3a*^R878H^ can confer impaired differentiation but not the complete differentiation block that is observed upon *Dnmt3a* loss [16], and *Dnmt3a*^R878H^ HSCs, unlike *Dnmt3a*^−/−^ HSCs [9, 16], do not appear to have overt lineage bias in the context of competitive transplantation.

In experiments examining the hematopoietic stem and progenitor cell compartment at different stages along the trajectory of CH to MPD and finally to AML, we revealed that *Npm1*^cA/+^ drives progressive transition of expansion of the HSC and MPP compartments in CH to the myeloid-restricted progenitor cell compartment in AML. Furthermore, these myeloid-restricted progenitor cells represent LSCs capable of propagating AML in transplanted recipient mice. Seminal work examining the pre-leukemic state in AML has suggested that the cell of origin acquiring *DNMT3A* mutation is an HSC, which causes an expanded pool of HSCs and downstream progenitors, within which additional mutations including *NPM1*^c^ are acquired to drive progression to AML [8]. These findings pointed to GMPs and/or mixed lineage progenitors (MLP) as the likely populations in which *NPM1*^c^ was acquired. This model is consistent with our findings that progression of *Dnmt3a*-mutant CH to *Dnmt3a-* and *Npm1*-mutant AML is accompanied by a depletion of the expanded pool of HSCs observed in CH and a shift towards an expanded pool of myeloid-restricted progenitors that possess LSC activity. These data also provide important context for interpretation of a previously published report demonstrating that conditional knockin of *Dnmt3a*^R878H^ initiates AML in the absence of other engineered mutations after a long latency [10]. This study found that LTHSCs decreased and myeloid lineage-restricted progenitors were expanded in their *Dnmt3a*-mutant AML mice. Based on these data taken together, we propose that a decline in HSC frequency and shift toward myeloid-restricted progenitor expansion in the HSPC compartment may serve as a biomarker for risk or early progression of CH to AML. Our data suggest that distinct cooperating mutations, in addition to *Npm1*, drive progression of *Dnmt3a*-mutant CH to MDS/MPD, MPD and AML. Mutations in the tumor suppressor *Cux1* and RNA splicing factor mutations are associated with MDS/MPD, while strong selection for mutations activating Ras/Raf/MAPK signaling are invariably associated with progression to MPD. Furthermore, transformation to AML is accompanied by selection for additional mutations in signaling molecules and/or epigenetic regulatory factors.

Mutations in *DNMT3A* are common in the general population and increase with aging. With next-generation sequencing becoming more and more routine as a part of general medical care, we need better predictive tools to assess who is at risk for progression from CH to MDS, MPN or AML in addition to development of new preventative therapeutic strategies. Addressing a number of these pressing basic and translational research questions regarding the evolution of CH to AML will now be possible by utilizing the in vivo models we have developed. For example, different stressors and environmental factors (age, tobacco use, prior radiation therapy) have been hypothesized to facilitate emergence of clinically relevant phenotypes of the *DNMT3A* mutation [38]. Our models will not only permit prospective testing of the effects of stress or environmental factors on the *Dnmt3a*-mutant CH phenotype, but also whether effects on CH ultimately change the risk of progression to malignancy upon acquisition of a cooperating mutation in *Npm1*. Furthermore, orthogonal regulation of the *Dnmt3a* and *Npm1* mutations will permit interrogation of the underlying biological mechanisms by which these mutations interact to cause malignancy, including hematopoietic cell-intrinsic interactions (ex. chromatin regulation, DNA repair) and cell-extrinsic interactions (ex. alterations in the BM cytokine environment). Lastly, this study serves as a proof-of-principle that utilizing an inducible, dual-recombinase system is a feasible and translationally relevant strategy to model combinations of somatic mutations in CH, pre-leukemia and hematologic malignancy, and to model clonal evolution more broadly in different malignant contexts.

## Supplementary information

Figure S1

Figure S2

Figure S3

Table S1

Table S2

Table S3
