# Supplementary figures and images for: Sequentially inducible mouse models reveal that Npm1 mutation causes malignant transformation of Dnmt3a-mutant clonal hematopoiesis
Source: Leukemia. 2019 Jan 28;33(7):1635–49. doi: 10.1038/s41375-018-0368-6 (PMC6609470; doi:10.1038/s41375-018-0368-6)

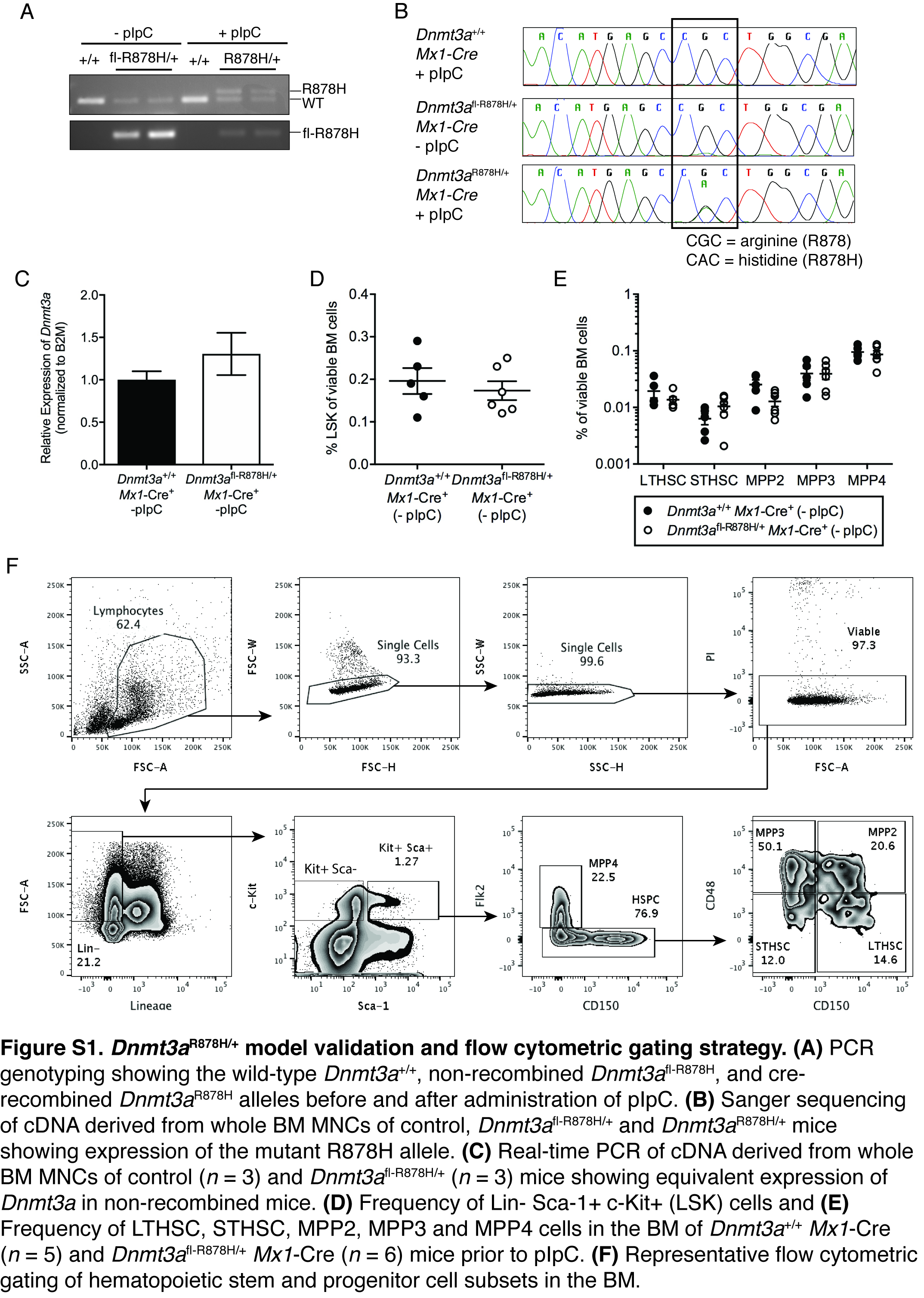

Supplement: Supplementary file 1 — Figure S1 [file 41375_2018_368_MOESM1_ESM.tif]

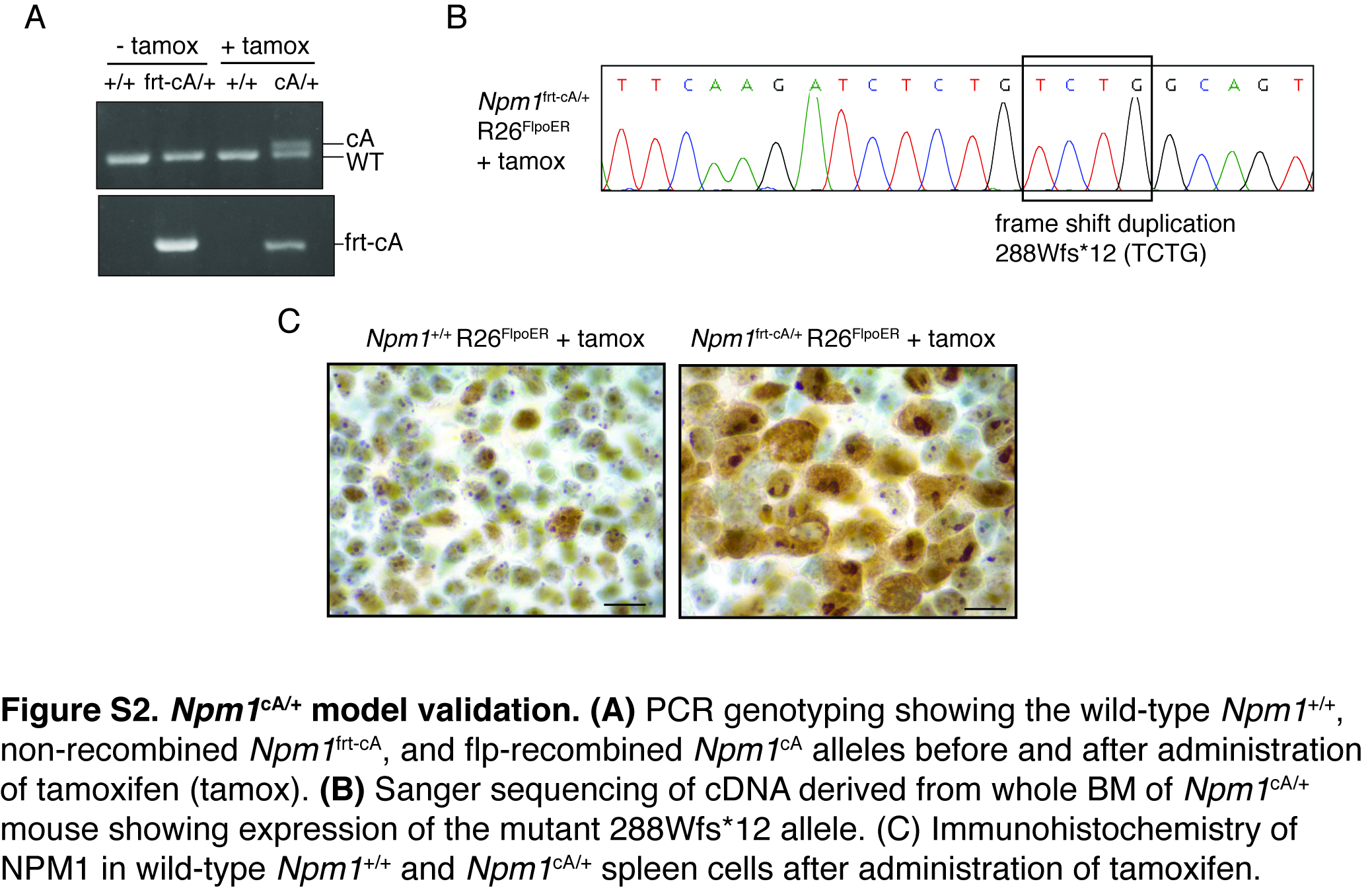

Supplement: Supplementary file 2 — Figure S2 [file 41375_2018_368_MOESM2_ESM.tif]
