# Supplementary material for: Sequentially inducible mouse models reveal that Npm1 mutation causes malignant transformation of Dnmt3a-mutant clonal hematopoiesis
Source: Leukemia. 2019 Jan 28;33(7):1635–49. doi: 10.1038/s41375-018-0368-6 (PMC6609470; doi:10.1038/s41375-018-0368-6)
Supplement: Supplementary file 3 — Figure S3 [file 41375_2018_368_MOESM3_ESM.pdf]

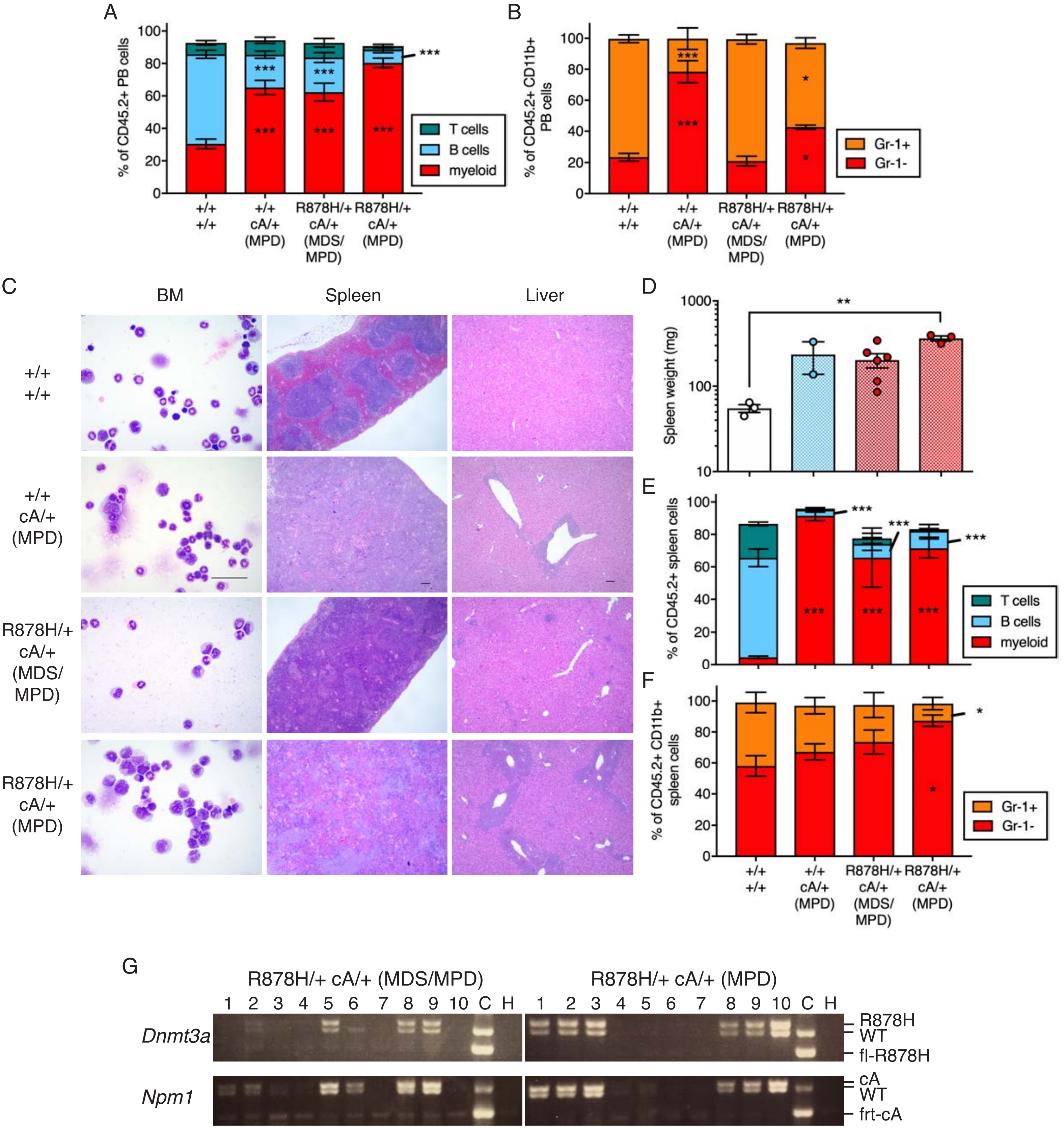

Phenotype	Frequency of recombined clones
MDS/MPD	82% (9/11)
MPD	100% (22/22)
AML	100% (15/15)

Figure S3. Characterization of *Dnmt3a*$^{R878H/+}$ *Npm1*$^{cA/+}$ MDS/MPD and MPD. (A) Frequency of myeloid, B and T cells within the donor-derived CD45.2^{+} fraction in PB and **(B)** Gr-1^{+} and Gr-1^{-} cells within donor-derived myeloid PB of moribund mice (control, *n* = 3; cA/+ MPD, *n* = 2; R878H/+ cA/+ MDS/MPD, *n* = 6; R878H/+ cA/+ MPD, *n* = 3). **(C)** Representative Giemsa-stained BM cytospin (far left; 40X, scale bars are 40um) and H&E-stained spleen (center; 4X, scale bars are 100um) and liver sections (far right; 4X, scale bars are 100um) from moribund recipient mice transplanted with control, cA/+ only or R878H/+ cA/+ BM cells. **(D)** Spleen weights of moribund mice (control, *n* = 3; cA/+ MPD, *n* = 2; R878H/+ cA/+ MDS/MPD, *n* = 6; R878H/+ cA/+ MPD, *n* = 3). **(E)** Frequency of myeloid, B and T cells within the donor-derived CD45.2+ fraction in PB and **(F)** Gr-1+ and Gr-1- cells within donor-derived myeloid PB of moribund mice (control, *n* = 3; cA/+ MPD, *n* = 2; R878H/+ cA/+ MDS/MPD, *n* = 10; R878H/+ cA/+ MPD, *n* = 3). **(G)** Representative gel images and summary of PCR genotyping of single CFU colonies derived from BM of R878H/+ cA/+ mice showing wild-type (WT), non-recombined fl-R878H or frt-cA, and recombined R878H or cA alleles. C: non-recombined control, H: water only control.
