# Supplementary material for: Sequentially inducible mouse models reveal that Npm1 mutation causes malignant transformation of Dnmt3a-mutant clonal hematopoiesis
Source: Leukemia. 2019 Jan 28;33(7):1635–49. doi: 10.1038/s41375-018-0368-6 (PMC6609470; doi:10.1038/s41375-018-0368-6)
Supplement: Supplementary file 4 — Table S1 [file 41375_2018_368_MOESM4_ESM.docx]

**Table S1. Primer sequences for genotyping and recombination PCR**

**Primer Name Application(s) Sequence (5’ to 3’)**

Dnmt3aR878H F1 genotyping, CCACTAGAACCCTCAGCACA

recombination

Dnmt3aR878H F2 genotyping, AGTAAGTCTGCAGGTCGAGG

recombination

Dnmt3aR878H R1 genotyping, CCCCAGACCTTTGAAATGCC

recombination

Npm1cA F1 genotyping, CAAAAGTGGTTAGGATTGAGGCAG

recombination

Npm1cA R1 genotyping, TAGCCTGAAGAACGAGATCAGC

recombination

Npm1cA R2 genotyping, CCTGTGGGTCAGCCCTATTT

recombination

Dnmt3a RT F real-time PCR ACTTGGAGAAGCGGAGTGAA

Dnmt3a RT R real-time PCR CTGTTCTTTGCCCTCTCCTG

B2M RT F real-time PCR TTCACCCCCACTGAGACTGAT

B2M RT R real-time PCR GTCTTGGGCTCGGCCATA
