# Supplementary material for: Sequentially inducible mouse models reveal that Npm1 mutation causes malignant transformation of Dnmt3a-mutant clonal hematopoiesis
Source: Leukemia. 2019 Jan 28;33(7):1635–49. doi: 10.1038/s41375-018-0368-6 (PMC6609470; doi:10.1038/s41375-018-0368-6)
Supplement: Supplementary file 5 — Table S2 [file 41375_2018_368_MOESM5_ESM.docx]

**Table S2. List of included genes in MSK Mouse-IMPACT**

| *Abl1* | *Cd274* | *Erbb3* | *Hist1h1c* | *Lmo1* | *Pak7* | *Rb1* | *Spry4* |
| --- | --- | --- | --- | --- | --- | --- | --- |
| *Actg1* | *Cd276* | *Erbb4* | *Hist1h1d* | *Ltb* | *Palb2* | *Rbm10* | *Src* |
| *Acvr1* | *Cd28* | *Ercc2* | *Hist1h1e* | *Lyn* | *Park2* | *Recql* | *Srsf2* |
| *Ago2* | *Cd48* | *Ercc3* | *Hist1h2ad* | *Malt1* | *Parp1* | *Recql4* | *Stag1* |
| *Akt1* | *Cd79a* | *Ercc4* | *Hist1h2ae* | *Map2k1* | *Pax5* | *Rel* | *Stag2* |
| *Akt2* | *Cd79b* | *Ercc5* | *Hist1h2an* | *Map2k2* | *Pbrm1* | *Ret* | *Stat3* |
| *Akt3* | *Cdc42* | *Erf* | *Hist1h2ao* | *Map2k4* | *Pcbp1* | *Rfwd2* | *Stat5a* |
| *Alk* | *Cdc73* | *Erg* | *Hist1h2bf* | *Map3k1* | *Pdcd1* | *Rheb* | *Stat5b* |
| *Alox12b* | *Cdh1* | *Errfi1* | *Hist1h2bg* | *Map3k13* | *Pdcd1lg2* | *Rhoa* | *Stat6* |
| *Amer1* | *Cdk12* | *Esco1* | *Hist1h2bk* | *Map3k14* | *Pdgfra* | *Rictor* | *Stk11* |
| *Ankrd11* | *Cdk4* | *Esco2* | *Hist1h2bp* | *Mapk1* | *Pdgfrb* | *Rit1* | *Stk19* |
| *Apc* | *Cdk6* | *Esr1* | *Hist1h3a* | *Mapk3* | *Pdpk1* | *Rnf43* | *Stk40* |
| *Ar* | *Cdk8* | *Etnk1* | *Hist1h3b* | *Mapkap1* | *Pds5a* | *Robo1* | *Sufu* |
| *Araf* | *Cdkn1a* | *Etv1* | *Hist1h3c* | *Max* | *Pds5b* | *Ros1* | *Suz12* |
| *Arhgef28* | *Cdkn1b* | *Etv6* | *Hist1h3d* | *Mcl1* | *Pgr* | *Rps6ka4* | *Syk* |
| *Arid1a* | *Cdkn2a* | *Ezh1* | *Hist1h3e* | *Mdc1* | *Phf6* | *Rps6kb2* | *Tap1* |
| *Arid1b* | *Cdkn2b* | *Ezh2* | *Hist1h3g* | *Mdm2* | *Phox2b* | *Rptor* | *Tap2* |
| *Arid2* | *Cdkn2c* | *Fam175a* | *Hist1h3h* | *Mdm4* | *Piga* | *Rragc* | *Tbl1xr1* |
| *Arid3a* | *Cebpa* | *Fam46c* | *Hist1h3i* | *Med12* | *Pik3c2g* | *Rras* | *Tbx3* |
| *Arid3b* | *Cenpa* | *Fam58b* | *Hist2h2be* | *Mef2b* | *Pik3c3* | *Rras2* | *Tceb1* |
| *Arid3c* | *Chek1* | *Fanca* | *Hist2h3b* | *Men1* | *Pik3ca* | *Rtel1* | *Tcf3* |
| *Arid4a* | *Chek2* | *Fancc* | *Hist2h3c1* | *Met* | *Pik3cb* | *Runx1* | *Tcf7l2* |
| *Arid4b* | *Cic* | *Fancd2* | *Hist2h3c2* | *Mga* | *Pik3cd* | *Runx1t1* | *Tek* |
| *Arid5a* | *Ciita* | *Fas* | *Hist3h2ba* | *Mgam* | *Pik3cg* | *Rxra* | *Tert* |
| *Arid5b* | *Crbn* | *Fat1* | *Hnf1a* | *Mitf* | *Pik3r1* | *Rybp* | *Tet1* |
| *Asxl1* | *Crebbp* | *Fbxo11* | *Hoxb13* | *Mlh1* | *Pik3r2* | *Samhd1* | *Tet2* |
| *Asxl2* | *Crkl* | *Fbxw7* | *Hras* | *Mob3b* | *Pik3r3* | *Sdha* | *Tet3* |
| *Atm* | *Crlf2* | *Fgf15* | *Icosl* | *Mpeg1* | *Pim1* | *Sdhaf2* | *Tgfbr1* |
| *Atp6ap1* | *Csde1* | *Fgf3* | *Id3* | *Mpl* | *Plcg1* | *Sdhb* | *Tgfbr2* |
| *Atp6v1b2* | *Csf1r* | *Fgf4* | *Idh1* | *Mre11a* | *Plcg2* | *Sdhc* | *Tmem127* |
| *Atr* | *Csf3r* | *Fgfr1* | *Idh2* | *Msh2* | *Plk1* | *Sdhd* | *Tmprss2* |
| *Atrx* | *Ctcf* | *Fgfr2* | *Ifngr1* | *Msh3* | *Plk2* | *Sesn1* | *Tnfaip3* |
| *Atxn2* | *Ctla4* | *Fgfr3* | *Igf1* | *Msh6* | *Pmaip1* | *Sesn2* | *Tnfrsf14* |
| *Aurka* | *Ctnnb1* | *Fgfr4* | *Igf1r* | *Msi1* | *Pms1* | *Sesn3* | *Top1* |
| *Aurkb* | *Cul3* | *Fh1* | *Igf2* | *Msi2* | *Pms2* | *Setbp1* | *Traf2* |
| *Axin1* | *Cux1* | *Flcn* | *Ikbke* | *Mst1* | *Pnrc1* | *Setd1a* | *Traf3* |
| *Axin2* | *Cxcr4* | *Flt1* | *Ikzf1* | *Mst1r* | *Pold1* | *Setd1b* | *Traf5* |
| *Axl* | *Cyld* | *Flt3* | *Ikzf3* | *Mtor* | *Pole* | *Setd2* | *Traf7* |
| *B2m* | *Cysltr2* | *Flt4* | *Il10* | *Mutyh* | *Pot1a* | *Setd3* | *Trp53* |
| *Babam1* | *Daxx* | *Foxa1* | *Il7r* | *Myc* | *Pparg* | *Setd4* | *Trp53bp1* |
| *Bach2* | *Dcun1d1* | *Foxl2* | *Inha* | *Mycl* | *Ppm1d* | *Setd5* | *Trp63* |
| *Bap1* | *Ddr2* | *Foxo1* | *Inhba* | *Mycn* | *Ppp2r1a* | *Setd6* | *Tsc1* |
| *Bard1* | *Ddx3x* | *Foxp1* | *Inpp4a* | *Myd88* | *Ppp4r2* | *Setd7* | *Tsc2* |
| *Bbc3* | *Dicer1* | *Fubp1* | *Inpp4b* | *Myod1* | *Ppp6c* | *Setd8* | *Tshr* |
| *Bcl10* | *Dis3* | *Furin* | *Inppl1* | *Nbn* | *Prdm1* | *Setdb1* | *Tyk2* |
| *Bcl11b* | *Dnajb1* | *Fyn* | *Insr* | *Ncoa3* | *Prdm14* | *Setdb2* | *U2af1* |
| *Bcl2* | *Dnmt1* | *Gata1* | *Irf1* | *Ncor1* | *Prex2* | *Sf3b1* | *U2af2* |
| *Bcl2l1* | *Dnmt3a* | *Gata2* | *Irf4* | *Ncor2* | *Prkar1a* | *Sgk1* | *Ubr5* |
| *Bcl2l11* | *Dnmt3b* | *Gata3* | *Irf8* | *Ncstn* | *Prkci* | *Sh2b3* | *Upf1* |
| *Bcl6* | *Dot1l* | *Gli1* | *Irs1* | *Negr1* | *Prkd1* | *Sh2d1a* | *Vav1* |
| *Bcor* | *Drosha* | *Gm10499* | *Irs2* | *Nf1* | *Ptch1* | *Shoc2* | *Vav2* |
| *Bcorl1* | *Dtx1* | *Gm12657* | *Jak1* | *Nf2* | *Pten* | *Shq1* | *Vegfa* |
| *Bcr* | *Dusp1* | *Gna11* | *Jak2* | *Nfe2* | *Ptp4a1* | *Slx4* | *Vhl* |
| *Birc3* | *Dusp22* | *Gna12* | *Jak3* | *Nfe2l2* | *Ptpn1* | *Smad2* | *Vtcn1* |
| *Blm* | *Dusp4* | *Gna13* | *Jarid2* | *Nfkbia* | *Ptpn11* | *Smad3* | *Wapl* |
| *Bmpr1a* | *E2f3* | *Gnaq* | *Jun* | *Nipbl* | *Ptpn2* | *Smad4* | *Whsc1* |
| *Braf* | *Eed* | *Gnas* | *Kdm5a* | *Nkx2-1* | *Ptprd* | *Smarca4* | *Whsc1l1* |
| *Brca1* | *Egfl7* | *Gnb1* | *Kdm5c* | *Nkx3-1* | *Ptprs* | *Smarcb1* | *Wt1* |
| *Brca2* | *Egfr* | *Gps2* | *Kdm6a* | *Notch1* | *Ptprt* | *Smarcd1* | *Wwtr1* |
| *Brd4* | *Egr1* | *Grem1* | *Kdr* | *Notch2* | *Rab35* | *Smc1a* | *Xbp1* |
| *Brip1* | *Eif1a* | *Grin2a* | *Keap1* | *Notch3* | *Rac1* | *Smc3* | *Xiap* |
| *Btg1* | *Eif4a2* | *Gsk3b* | *Kit* | *Notch4* | *Rac2* | *Smg1* | *Xpo1* |
| *Btk* | *Eif4e* | *Gtf2i* | *Klf4* | *Npm1* | *Rad21* | *Smo* | *Xrcc2* |
| *Calr* | *Elf3* | *H2-Q2* | *Kmt2a* | *Nras* | *Rad50* | *Smyd3* | *Yap1* |
| *Card11* | *Ep300* | *H3f3a* | *Kmt2b* | *Nsd1* | *Rad51* | *Socs1* | *Yes1* |
| *Carm1* | *Ep400* | *H3f3c* | *Kmt2c* | *Nt5c2* | *Rad51b* | *Sos1* | *Zfhx3* |
| *Casp8* | *Epas1* | *Hdac1* | *Kmt2d* | *Nthl1* | *Rad51c* | *Sox17* | *Zrsr2* |
| *Cbfb* | *Epcam* | *Hdac4* | *Knstrn* | *Ntrk1* | *Rad51d* | *Sox2* |  |
| *Cbl* | *Epha3* | *Hdac7* | *Kras* | *Ntrk2* | *Rad52* | *Sox9* |  |
| *Ccnd1* | *Epha5* | *Hdac8* | *Ksr2* | *Ntrk3* | *Rad54l* | *Sp140* |  |
| *Ccnd2* | *Epha7* | *Hgf* | *Lats1* | *Nuf2* | *Raf1* | *Spen* |  |
| *Ccnd3* | *Ephb1* | *Hif1a* | *Lats2* | *Nup93* | *Rara* | *Spop* |  |
| *Ccne1* | *Erbb2* | *Hist1h1b* | *Lck* | *Pak1* | *Rasa1* | *Spred1* |  |
